# Optical characterization of epidermal cells and their relationship to DNA recovery from touch samples

**DOI:** 10.12688/f1000research.7385.1

**Published:** 2015-11-26

**Authors:** Cristina E. Stanciu, M. Katherine Philpott, Ye Jin Kwon, Eduardo E. Bustamante, Christopher J. Ehrhardt

**Affiliations:** 1 Department of Forensic Science, Virginia Commonwealth University, Richmond, VA, 23284, USA

**Keywords:** forensic science, flow cytometry, extracellular DNA, epidermal cell, touch DNA

## Abstract

The goal of this study was to investigate the relative contributions of different cellular and genetic components to biological samples created by touch or contact with a surface – one of the most challenging forms of forensic evidence. Touch samples were generated by having individuals hold an object for five minutes and analyzed for quantity of intact epidermal cells, extracellular DNA, and DNA from pelleted cell material after elution from the collection swab. Comparisons were made between samples where individuals had washed their hands immediately prior to handling and those where hand washing was not controlled. The vast majority (84-100%) of DNA detected in these touch samples was extracellular and was uncorrelated to the number of epidermal cells detected. Although little to no extracellular or cell pellet-associated DNA was detected when individuals washed their hands prior to substrate handling, we found that a significant number of epidermal cells (between ~5x10 ^3^ and ~1x10 ^5^) could still be recovered from these samples, suggesting that other types of biological information may be present even when no amplifiable nuclear DNA is present. These results help to elucidate the biological context for touch samples and characterize factors that may contribute to patterns of transfer and persistence of genetic material in forensic evidence.

## Introduction

‘Touch’ or trace DNA samples represent a significant portion of evidence submitted to forensic caseworking laboratories. Understanding the mechanisms of DNA transfer through touch and developing methods to maximize the level of DNA recovery from contact surfaces is a continuing priority for the forensic science community ^1^. Historically, the quantity of DNA found in a contact sample was thought to be primarily based on the number of cells that people shed naturally from the outermost layer of skin ^2^. This concept continues to be perpetuated in the forensic community and analysts still testify to this effect ^3– 5^.

However, recent studies have shown that touch samples can also contain ‘cell-free’ or extracellular nucleic acids (referred to as CNAs, eDNA, or cfDNA; in contrast to intracellular DNA or iDNA) that could be derived from a variety of sources such as sweat and oil secretions ^6– 10^. Additionally, it has been suggested that small amounts of saliva can be transferred through touch which may be a source of both cell-free DNA and intracellular DNA (via nucleated buccal cells) to a contact sample ^11^.

Although there are many possible sources of genetic material in touch evidence, the proportion of cellular and extracellular components is currently unclear. A recent survey of casework samples reported that more than 70% of contact samples contained extracellular DNA, which often provided an added value to the short tandem repeat (STR) profile generated from the pelleted cellular material ^6^. The study also found that the relative proportion of extracellular DNA to the total amount of DNA in each sample varied considerably.

In addition to understanding their relative contributions to contact samples, the forensic community would also benefit from determining whether different factors affect the deposition and persistence of epidermal cells and extracellular DNA on touched surfaces. Addressing these issues has important implications for optimizing DNA collection techniques as well as developing alternative analytical strategies for processing caseworking samples (e.g., 12).

Therefore, the goal of this study was to investigate the relative contributions of extracellular and intracellular DNA and their relationship to the quantity of cells recovered from touch samples under controlled conditions, and assess how the transfer and recovery of each type of biological material may be influenced by particular actions of the individual contributor. To accomplish this, we used flow cytometry for precise and non-destructive measurements of touch samples that were simultaneously processed using standard caseworking techniques for DNA analyses.

## Methods

### Sample collection

For initial imaging studies, two individuals were asked to hold a sterile conical tube (P/N: 229421; Celltreat Scientific) in one hand for five minutes. Samples were collected from the tube surface with one sterile, pre-wetted swab (P/N: 22037924; Fisher Scientific) followed by one dry swab. To elute the cells into solution, the swabs were manually stirred then vortexed for 15 seconds in 1 mL of Sterile DNAse-Free, Protease-Free Water (P/N: BP24701; Fisher Scientific). All procedures for participant solicitation and consent for human subject research were approved by the VCU-Institutional Review Board (ID# HM20000454_CR).

For experiments involving comparisons of cell and DNA yields between washed and unwashed hands, two sets of two samples (one tube in each hand) were collected from eight individuals using the protocol described above: the first set of 16 was collected before hand washing, and the second after washing hands with soap and water for 20 seconds and air drying. A 20μL aliquot of each 1mL cell solution was used in subsequent flow studies (including cell enumeration), and the remaining 980μL was used for DNA studies.

Another 20 samples were collected without any control for hand washing from these eight donors, along with three additional donors, using the protocol described above. The entirety of each of these samples was processed for DNA.

### Microscopic imaging

**Figure 1.**
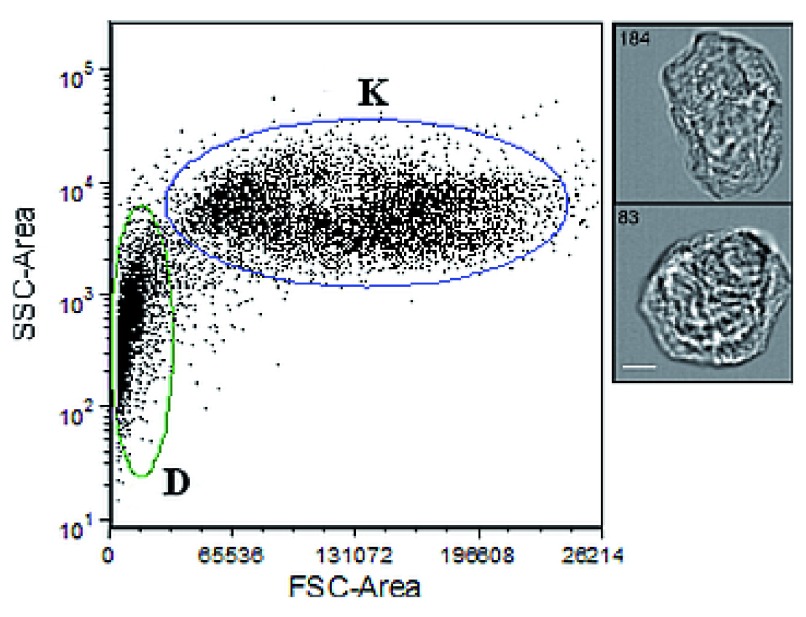
Optical characterization of a touch sample. Cell events fall into two distinct populations along the Forward Scatter (FSC) and Side Scatter (SSC) axes: intact cells (‘K’) and cell debris (‘D’). Right insets show images of individual events within the K population. Scale bar=7 µm.

In order to separate intact cells from debris and cellular fragments for imaging purposes, after passing cell suspensions through a 100 µm mesh filter, Fluorescence-Activated Cell Sorting (FACS) was performed on the BD FACSAria™ Ilu (Becton Dickinson) flow cytometer using a 488 ηm Coherent solid-state laser. Channel voltages were set as follows: FSC, 50V; SSC, 200V. Events falling into gate “K” (see Figure 1) were sorted into a new tube, then imaged using the Amnis ^®^ Imagestream X MK II Software (EMD Millipore) by activating the Bright Field channel. Pictures were analyzed and exported with the IDEAS ^®^ Software v6.1 (EMD Millipore).

### Flow cytometry analysis and cell enumeration

Cell suspensions were passed through a 100 µm mesh filter prior to flow cytometry analysis on the BD FACSCanto™ II analyzer (Becton Dickinson) using 488 ηm and 633 ηm lasers. The channel voltages were set as follows: FSC, 150V; SSC, 200V; FITC, 335V; PE, 233V; PE-Cy5, 300V; PE-Cy7, 400V; and APC, 250V. Data acquisition was performed using the FACSDIVA Software v8.0.1 (Becton Dickinson) and analyzed using FCS Express 4.0 (DeNovo).

In order to precisely quantify the cells in our samples during flow analysis, we spiked our cell solutions with a known concentration of 123 eBeads (01-1234-42; Affymetrix eBioscience), fluorescently-labeled microparticle standards that are 7 µm and easily distinguishable from our target cell population both in size and fluorescence (FITC, PE, and APC channels). The ratio of cells to beads was then used to determine the concentration of cells in the sample through the following formula:

$$\begin{array}{l} \text{Absolute Count}\;(\text{cells}/\mu \text{L})=\underline{\left(\text{Cell Count}\;\times \;\text{eBead Volume})(\text{eBead Concentration}\right)}\\ \;(\text{eBead Count}\;\times \;\text{Cell Volume}) \end{array}$$

The concentration of cells in the 20µL aliquot was then used to estimate the total number of cells present in the entire volume of eluent (1mL) recovered from the collection swabs. Flow cytometry analysis of bead standards was conducted on events detected within the ‘K’ gate for each sample. The procedures for detecting and differentiating eBeads from target cells followed the manufacturer's suggested protocol ( http://www.ebioscience.com/media/pdf/tds/01/01-1234.pdf).

### Isolation and extraction of extracellular and cell pellet DNA

Once an aliquot was removed for cell quantification, the remaining cell suspension was transferred to a new 2mL collection tube and centrifuged at 10,000 xg for five minutes at room temperature. The supernatant was added to a pre-washed Amicon filter (UFC210024; EMD Millipore). The remaining pellet was washed with 500 µL of sterile water twice, each time adding the supernatant to the Amicon filter. The combined supernatant fraction was then centrifuged at 3,220 ×g for 30 minutes, followed by a wash step in 2 mL 1xTE Buffer (P/N 50-843-203; Teknova). The DNA was collected by inverting the filter and centrifuging at 1,000 xg for 2 minutes at room temperature. The final volume of the eluted retentate was approximately 20 µl.

Additionally, in order to maximize the recovery of cell material and/or DNA, the wet swab tips were placed in a spin basket (P/N 19597; Investigator Lyse & Spin Basket Kit; Qiagen) immediately following the initial elution, and centrifuged at 10,000 ×g for 5 minutes at room temperature (absent any additional reagents). The resulting liquid eluent that passed through the spin basket was added to the supernatant solution (prior to Amicon filtration) and remaining cell pellet in the spin basket was dissolved in ~50 µl of sterile water, combined with its respective fraction, and subjected to DNA extraction using the following protocol. The cell pellet material was incubated with 500 µL Cell Lysis Buffer (P/N BDB559759; BD Pharmigen) and 10 µL Proteinase K (P/N EO0491; Fisher Scientific) in a 56°C water bath for 17 hours. The sample was then centrifuged at 10,000 ×g for 5 minutes at room temperature. The supernatant was purified with an equal volume of UltraPure Phenol:Chloroform:Isoamyl Alcohol (P/N 15593-031; Life Technologies (25:24:1, v/v)), then 1xTE Buffer, and finally concentrated to a final volume of 20–40 µL using a pre-washed Amicon filter.

### DNA quantitation

DNA quantitation was performed using the Investigator Quantiplex ^®^ Human Kit (P/N 387016, Qiagen) coupled with the ABI Prism 7500 Sequence Detection System (Applied Biosystems). A 25 µl reaction was used for all samples following manufacturer’s suggested protocol (‘Investigator Quantiplex Handbook’, www.qiagen.com).

## Results

Initial characterizations of touch samples with flow cytometry showed two distinct size fractions (‘K’ and ‘D’ populations in Figure 1). Size and morphological information derived from AMNIS images of the K fraction from two touch samples revealed that this population was consistent with fully differentiated keratinocytes ( *i.e.*, corneocytes) ~20–40 µm in diameter, while the D fraction was consistent with cellular debris/fragments. Other epithelial cell types ( *e.g.*, buccal cells >60 µm) were not observed among the AMNIS images captured ( Figure S1, Figure S2).

Cell counts and DNA yields were compared across 31 touch samples generated from eight different individuals that used both dominant and non-dominant hands to hold the substrate. To investigate the effect of hand washing on the transfer of cellular and extracellular components of a touch sample, half of these samples were collected after donors had washed their hands and the other half without immediate hand washing.

**Figure 2.**
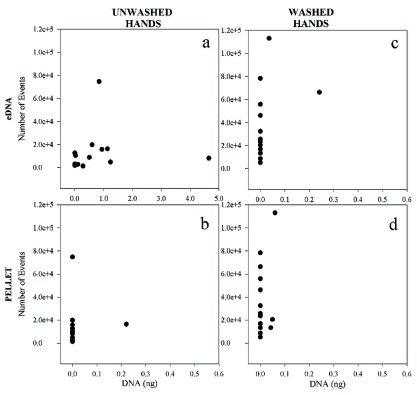
Cell counts and DNA yields from touch samples from washed and unwashed hands. For each graph, the Y axis represents the number of “K events” (cells) detected in solution from collection swabs (unwashed hands in **a** and **b**; washed hands in **c** and **d**), while the X axis represents the number of nanograms of DNA recovered (from supernatant ( **a**) and cell pellet ( **b**) of unwashed hands, and from supernatant ( **c**) and cell pellet ( **d**) of washed hands).

An estimated ~5×10 ^3^ to ~1×10 ^5^ cells were recovered from washed hand samples, versus ~1×10 ^3^ to ~8×10 ^4^ cells from unwashed hand samples ( Figure 2; Table S1). Overall, we observed greater transfer of cells in the washed hand samples than the unwashed hand samples (median of 2.5×10 ^4^ cells vs. 8.6×10 ^3^ cells, respectively). Despite the often high recovery of cells from touch samples, DNA recovery from the cell pellet was consistently low, whether from washed or unwashed hands. DNA was detected in the cell pellet of one unwashed hand sample (0.220 ng) and three washed hands samples (0.049, 0.042, 0.060 ng). No DNA was detected in any of the other cell pellets.

In contrast, consistent differences were observed in eDNA recovery from samples generated from washed versus unwashed hands. Little to no DNA was recovered from the extracellular fraction of touch samples left by donors who had washed their hands, with quantitation values ranging from zero to 0.242 ng ( Figure 2c). In samples from unwashed hands, extracellular DNA recovery varied between zero and 4.646 ng ( Figure 2a). There was no apparent correlation between the number of cells and the quantity of DNA recovered from the samples (either eDNA or cell pellet). Neither could DNA recovery with or without hand washing be correlated to hand dominance, in contrast to findings by others ^13^.

The additional 20 samples tested for relative quantity of eDNA versus intracellular DNA produced results that are consistent with the above findings ( Table 1, compilation of all samples without hand washing (n = 35)). In samples where DNA was detected, the total proportion of eDNA ranged from 84–100% with the majority of the samples at or near 100%.

**Table 1.** Proportion of DNA in supernatant and in cell pellet after three washes.

Sample	Extracellular DNA (ng)	DNA in Cell Pellet (ng)	Percentage eDNA
**D11**	0.607	ND	100
	1.191	0.068	95
	0.603	ND	100
	0.842	ND	100
	2.023	ND	100
**E14**	2.296	0.037	98
	1.134	0.220	84
	0.504	ND	100
	ND	ND	n/a
**E15**	2.162	0.284	88
	0.567	ND	100
	4.646	ND	100
	0.940	ND	100
	2.842	ND	100
**C81**	ND	ND	n/a
	ND	ND	n/a
	0.282	ND	100
**D02**	ND	ND	n/a
	0.374	ND	100
	ND	ND	n/a
	0.028	ND	100
	ND	ND	n/a
**H73**	ND	ND	n/a
	ND	ND	n/a
	0.780	ND	100
**I66**	0.286	ND	100
	1.240	ND	100
	1.110	ND	100
**J16**	1.804	0.021	99
	1.262	ND	100
**Y02**	0.058	ND	100
	0.106	ND	100
	0.314	ND	100
**K08**	ND	ND	n/a
**S07**	ND	ND	n/a

ND=below the limit of detection, ~1 pg/µl. Samples refer to individual donors. Each row within a single donor shows results from replicate experiments performed on different days.

## Discussion

Our results contribute to the forensic community’s growing body of knowledge on touch samples. We found that the vast majority (~84–100%) of nuclear DNA recovered from touch samples collected under the conditions described above is extracellular. Amplifiable DNA from the pelleted cellular fraction was detected in only eight of the 51 touch samples analyzed ( Figure 2, Table 1).

Although this finding is generally consistent with other recent studies suggesting the significance of extracellular DNA in touch evidence ^6, 8^, the prevalence and proportion of extracellular DNA relative to the total DNA yield shown in Table 1 was higher than observed in other studies ^6^. It is possible that the multiple wash steps performed on the pelleted cell material for this study removed more eDNA than efforts utilizing a single wash. In a separate analysis of seven replicate samples, we found that additional eDNA was often recovered with additional wash steps, and concurrently, that a clear systematic cell loss at each wash step was not observed—a Student’s t-test on cell counts before and after three wash steps yielded an average p-value of 0.28 with only two of the individual replicates yielding p-values less than 0.01 ( Table S2). This suggests that while some cells may have been unintentionally removed from some cell pellets by our methodology, this phenomenon is unlikely to explain the consistent increased DNA recovery in the supernatant with additional washes across samples.

The nature of the samples likely played a role as well, as there may have been more opportunities to pick up nucleated cells for some casework samples described in other research ^6^ than our controlled conditions. The fact that the “typical” or “standard” touch sample evades definition poses a challenge when designing studies to better understand these kinds of samples. It has been suggested that saliva, which contains buccal cells, may be an important ( *i.e.*, DNA rich) component of some touch samples ^11^. We observed no evidence of such cells – which generally appear larger than corneocytes (>60 µm for buccal cells versus 20–40 µm for corneocytes) – in microscopic surveys of individual cells within two touch samples ( Figure S1, Figure S2). However, this does not preclude the possibility that non epidermal cells were present, since only a portion of the sample was surveyed, and because deformed or fragmented cells from different tissues may be indistinguishable from corneocytes. Future work could explicitly test for the presence of buccal cells in touch samples through, e.g., antibody hybridizations targeting tissue specific surface antigens coupled with flow cytometry.

The mechanism of touching could also affect the proportion of eDNA to iDNA in touch samples; our preliminary data from touch samples deposited by rubbing suggest that this action may result in considerably higher cell pellet yields than samples deposited by holding, perhaps by exposing deeper (i.e., undifferentiated) layers of cells. However, in these preliminary experiments we also observed that the amount of eDNA left by rubbing the substrate was similar to levels of eDNA left by holding. This suggests that the transfer of eDNA may not be as affected by the manner in which a substrate was handled as iDNA transfer.

In any case, our results lend further support to the concept that extracellular DNA is particularly crucial to the analysis of touch samples. Measures should be explored to exploit this source of information to the greatest extent possible. For sample collection and processing purposes, this may dictate that touch samples be treated differently than other types of forensic biological sample. To avoid the significant loss of DNA that may be associated with extraction, it may make sense to process the eDNA-containing supernatant separately via direct amplification; our results suggest that care should be taken to maximize the amount of eDNA washed into the supernatant.

Our finding that the number of cells in touch samples was uncorrelated to the amount of extracellular DNA or the total DNA yield suggests that not only is the recoverable DNA primarily extracellular but that it is not immediately derived from the large numbers of epidermal cells that are shed daily. DNA was not detected in the cell pellet of samples that contained more than 100,000 cells, while samples comprised of far fewer cells (~2000) yielded DNA. Our extraction methodology likely had some impact on overall DNA yield ^14^; we have found in other experiments that other extraction methodologies (e.g., DNA IQ) resulted in low (<80pg) but quantifiable DNA yields in samples that yielded no DNA after processing with the extraction method utilized here. However, this does not change the fact that a considerable portion of DNA from the touch samples that we analyzed was extracellular, and that the number of cells shed was not a reliable indicator of DNA yield. These results are compatible with previous medical research showing that corneocytes from the outermost epidermal layer (i.e., *stratum corneum*) have little to no genomic DNA owing to the controlled degradation of intracellular components during differentiation ^15^.

Accordingly, epidermal cells – even when present in large quantities – may make a fairly insignificant contribution to either intra- or extracellular DNA recovery from touch samples. Consistent with recent studies that found no evidence of fragmented DNA in the epidermal layers (in contrast to sebaceous cell sources) ^10^, the majority of extracellular DNA in touch samples is likely derived from alternate sources such as oil and sweat secretions, or saliva ^8, 11^. Where intracellular (i.e., cell pellet) DNA levels from touch samples are considerably higher than those observed in this study, a nucleated cell source (i.e., non-epidermal, or more basal epidermal) may be implicated, though certain skin conditions are known to result in the aberrant retention of nuclear DNA in corneocytes ^15^.

Although hand washing resulted in the transfer and subsequent recovery of little to no eDNA, we found that cells were nonetheless transferred. In fact, we observed greater levels of cellular transfer among washed hand samples than unwashed hand samples. It is possible that the act of hand washing loosens or sloughs off corneocytes, and that these cells (perhaps because of their flattened morphology) are more likely to persist through the washing process than eDNA. Regardless of the explanation, an estimated thousands to hundreds of thousands of cells survived the hand washing process to be transferred from the palmar surface by simple touching.

Consistent with Locard’s principle, while these shed corneocytes may not contain sufficient levels of nuclear DNA to generate a probative STR profile, there is the possibility that other, non-genetic signatures could be analyzed, so that the most challenging touch samples (i.e. those that contain little to no DNA) may provide forensically relevant information. For example, the average size of individual corneocytes has been shown to vary with source factors such as age, sex, and anatomical region ^16– 18^, as does the composition of intracellular cytokeratin components ^19^. While further research is of course necessary to assess the degree of inter- and intra-individual variance in particular cellular features, determining such source attributes from unknown contributors could potentially provide leads or exclude suspects in specific types of investigations, e.g., sexual assault, molestation. Further, the absence of amplifiable nuclear DNA in corneocytes does not necessarily preclude the presence of sufficient levels of mitochondrial DNA to permit typing. Combining techniques to sort epidermal cells into donor populations (e.g., using factors described above) and typing the mtDNA of those populations is an avenue that warrants further exploration.

Overall, our observations suggest that many traditional explanations of DNA analysis from touch samples used in expert testimony – which often seek to explain the quantity and quality of DNA detected (or lack thereof) in terms of an individual’s inherent or circumstantial susceptibility to shed epidermal cells – may need to be modified to reflect fundamental shifts in the forensic community’s understanding of touch evidence. Future research efforts should continue to examine the relationship between the transfer of eDNA, iDNA, and intact corneocytes onto touch surfaces by testing other types of depositional circumstances, e.g., different substrate material or touch samples from multiple donors.

## Supplementary materials

**Figure S1. Supplemental imaging results for individual events within the large cell fraction of touch samples.** All cells were collected from the ‘K’ population shown in Figure 1. Scale bar=7 µm.

.

**Figure S2. Supplemental imaging results for individual events within the small cell fraction of touch samples.** All cells were collected from the ‘D’ population shown in Figure 1. Scale bar=7 µm.

.

**Table S1. Source data showing DNA yield and cell counts for all analyzed touch samples.** DNA quantitation and cell enumeration was performed as described in the Methods section. Unique sample names (e.g., ‘E14’) correspond to individual donors. ‘N/A’ indicates that cell counts were not performed on that sample. ‘ND’ indicates that DNA concentration was below the limit of detection, ~1pg/µl.

.

**Table S2. Source data showing cell counts from touch samples before and after three water wash steps.** Cell enumeration was performed using fluorescently labeled microparticle standards (See Methods). Unique sample names (e.g., ‘E14’) correspond to individual donors. Replicate experiments using the same donors are designated by 'exp1' and 'exp2'.
