# Supplementary figures and images for: Optical characterization of epidermal cells and their relationship to DNA recovery from touch samples
Source: F1000Res. 2015 Nov 26;4:1360. [Version 1] doi: 10.12688/f1000research.7385.1 (PMC4732551; doi:10.12688/f1000research.7385.1)

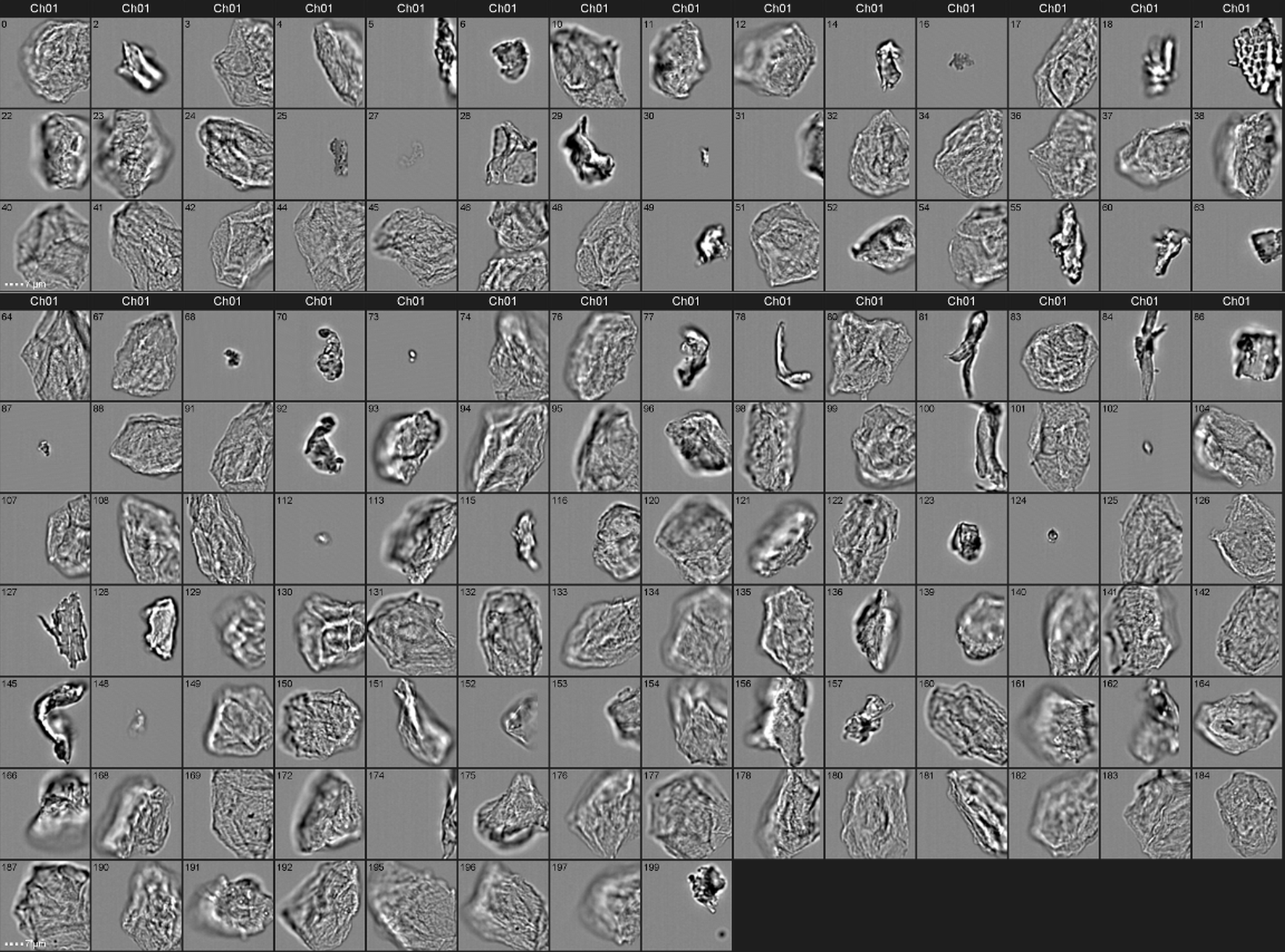

Supplement: Supplementary file 1 [file f1000research-4-7959-s0000.tgz › 1abd79cd-4b76-4054-adfd-47936d56aaff.tif]

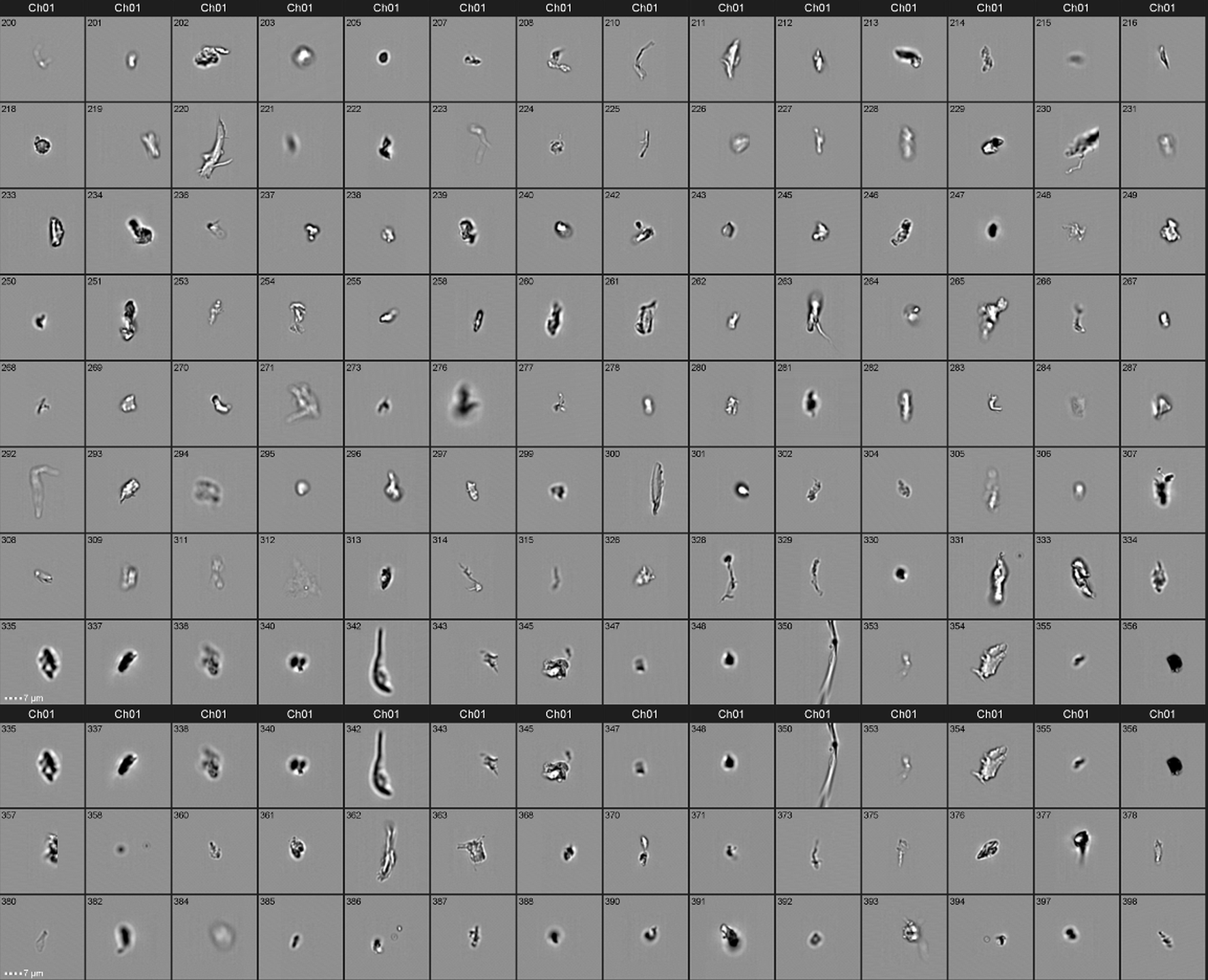

Supplement: Supplementary file 2 [file f1000research-4-7959-s0001.tgz › 6f774c33-ac12-400c-97a3-f902cc53cdbe.tif]
