# Supplementary material for: Optical characterization of epidermal cells and their relationship to DNA recovery from touch samples
Source: F1000Res. 2015 Nov 26;4:1360. [Version 1] doi: 10.12688/f1000research.7385.1 (PMC4732551; doi:10.12688/f1000research.7385.1)
Supplement: Peer review history

# Peer review: Referee response for version 1

**DOI:** 10.5256/f1000research.7959.r12080

**Published:** 2016-01-25

**Authors:** Antonio Filippini

**Affiliations:** 1 Department of Anatomy, Histology, Forensic Medicine, and Orthopedics, Sapienza University of Rome, Rome, Italy

The manuscript “Optical characterization of epidermal cells and their relationship to DNA recovery from touch samples” by Stanciu CE *et al*. is focused on the “Touch DNA” topic and is aimed at investigating the relative contributions of extracellular and intracellular DNA and their relationship to the quantity of cells recovered from touch samples under controlled conditions. It also assesses how the transfer and recovery of each type of biological material may be influenced by particular actions of the individual contributor.

To this aim, the Authors used flow cytometry for precise and non-destructive measurements of touch samples that were simultaneously processed using standard caseworking techniques for DNA analyses. The well described analytical methods and results show that there was no apparent correlation between the number of cells and the quantity of DNA recovered from the samples, neither could DNA recovery with or without hand washing be correlated to hand dominance.

According to recent studies, they found that the vast majority (~84–100%) of nuclear DNA recovered from touch samples collected under the conditions described above is extracellular (i.e. oil and sweat secretions, saliva), and suggest that future work could explicitly test for the presence of buccal cells in touch samples through, e.g., antibody hybridizations targeting tissue specific surface antigens coupled with flow cytometry.

They also highlighted the peculiarities, and consequently the analytical challenges, of touch samples, thus suggesting these samples to be treated differently than other types of forensic biological sample as for sample collection and processing purposes; e.g. to avoid the significant loss of DNA that may be associated with extraction, it may make sense to process the eDNA-containing supernatant separately via direct amplification.

An interesting and innovative element represented in the manuscript is the possibility that non-genetic signatures could be analyzed, so that the most challenging touch samples (i.e. those that contain little to no DNA) may provide forensically relevant information. To this regard, it is reported that “… the average size of individual corneocytes has been shown to vary with source factors such as age, sex, and anatomical region, as does the composition of intracellular cytokeratin components … combining techniques to sort epidermal cells into donor populations (e.g., using factors described above) and typing the mtDNA of those populations is an avenue that warrants further exploration …”.  

Finally, as an interested researcher in the field of “Touch DNA”, I appreciate the efforts of the Authors in preparing this interesting manuscript, that is well written and follows a logical structure. I just have a minor suggestion to improve the manuscript, that is to include some STR profiles in order to show the quality of the DNA recovered from the analyzed samples.

I have read this submission. I believe that I have an appropriate level of expertise to confirm that it is of an acceptable scientific standard.

# Peer review: Referee response for version 1

**DOI:** 10.5256/f1000research.7959.r11361

**Published:** 2015-12-10

**Authors:** Ram Gopal

**Affiliations:** 1 Department of Cell and Molecular Biology, Uppsala University, Uppsala, Sweden

The manuscript ‘’Optical characterization of epidermal cells and their relationship to DNA recovery from touch samples’’ by Stanciu CE *et al.* describes the genetic (nuclear DNA) and biological components (Cells and Cell debris) in samples created by touch or contact with a surface, both in controlled and non controlled situations. The question to understand and characterize the components of touch sample is of paramount importance in forensic science research. The manuscript attempts to decipher the mechanism of DNA transfer in touch samples and suggests methods to maximize the recovery of DNA from the touch samples. 

On the cellular components side, the FACS coupled with microscopy technique was employed to explore the number and identity of  the cells in touch samples. The cells in touch samples were found to be consistent with the keratinocyte morphology and size. This work also shows that after washing hands there is tendency to shed more cells in touch samples but the increase in the cell number is not correlated with the increase in DNA yield, rather there was no or very little DNA recovered after washing hands. Thus, this study provides evidence that there is no or very little DNA associated with the cellular component of the touch samples. It makes a strong case for the next important question to ask. How we can differentiate keratinocytes from different individuals? 

As the authors suggests that other biological information still may be present on the keratinocytes, it opens up the possibilities of a new field for characterization of keratinocytes from touch samples. The manuscript’s conclusion that the source of majority of DNA in touch samples comes from extracellular components, not from the cellular components, is in well agreement of previous studies but the proportion of extracellular DNA is found to be higher in this study than the earlier reports. The manuscript is well written and presents the data in a logical way. Overall, the manuscript adds further knowledge to the body of knowledge existing in this field and I recommend this manuscript for indexation.  

However I have following minor suggestions/comments to improve the manuscript.

1. No profiles, conventional or LCN, are shown to show the quality of DNA recovered.
2. It will be interesting to explore the contributions (both of DNA as well as Cells components) made by normal flora of human skin.
3. ‘’....flow cytometer using a 488 ηm Coherent solid-state laser.... ‘’ in this line and elsewhere the nanometer symbol should be written as ‘nm’.

I have read this submission. I believe that I have an appropriate level of expertise to confirm that it is of an acceptable scientific standard.
